# Tropifexor plus cenicriviroc combination versus monotherapy in nonalcoholic steatohepatitis: Results from the phase 2b TANDEM study

**DOI:** 10.1097/HEP.0000000000000439

**Published:** 2023-05-11

**Authors:** Quentin M. Anstee, Kathryn J. Lucas, Sven Francque, Manal F. Abdelmalek, Arun J. Sanyal, Vlad Ratziu, Adrian C. Gadano, Mary Rinella, Michael Charlton, Rohit Loomba, Edward Mena, Jörn M. Schattenberg, Mazen Noureddin, Donald Lazas, George B.B. Goh, Shiv K. Sarin, Yusuf Yilmaz, Miljen Martic, Rowan Stringer, Jossy Kochuparampil, Li Chen, Gerardo Rodriguez-Araujo, Elaine Chng, Nikolai V. Naoumov, Clifford Brass, Marcos C. Pedrosa

**Affiliations:** 1 Translational & Clinical Research Institute, Faculty of Medical Sciences, Newcastle University, Newcastle upon Tyne, UK; 2 Diabetes and Endocrinology Consultants, Morehead City, North Carolina, USA; 3 Department of Gastroenterology Hepatology, Antwerp University Hospital, Antwerp, Belgium; 4 InflaMed Centre of Excellence, Laboratory for Experimental Medicine and Paediatrics, Translational Sciences in Inflammation and Immunology, Faculty of Medicine and Health Sciences, University of Antwerp, Antwerp, Belgium; 5 European Reference Network on Hepatological Diseases (ERN RARE-LIVER); 6 Mayo Clinic, Rochester, Minnesota, USA; 7 Virginia Commonwealth University, Richmond, Virginia, USA; 8 Sorbonne Université, Hôpital Pitié Salpêtrière, ICAN Paris, France; 9 Liver Unit, Hospital Italiano de Buenos Aires, Argentina; 10 University of Chicago, Pritzker School of Medicine, Chicago, Illinois, USA; 11 University of Chicago, Chicago, Illinois, USA; 12 University of California at San Diego, La Jolla, California, USA; 13 California Liver Research Institute, Pasadena, California, USA; 14 Metabolic Liver Research Program, I. Department of Medicine, University Medical Center Mainz, Germany; 15 Houston Research Institute, Houston, Texas, USA; 16 Digestive Health Research and ObjectiveHealth, Nashville, Tennessee, USA; 17 Department of Gastroenterology and Hepatology, Singapore General Hospital, Singapore; 18 Department of Hepatology, Institute of Liver and Biliary Sciences, New Delhi, India; 19 Department of Gastroenterology, School of Medicine, Marmara University, Istanbul, Turkey; 20 Department of Gastroenterology, School of Medicine, Recep Tayyip Erdoğan University, Rize, Turkey; 21 Novartis Pharma AG, Basel, Switzerland; 22 Novartis Pharmaceuticals Corporation, East Hanover, New Jersey, USA; 23 AbbVie, California, USA; 24 HistoIndex Pte. Ltd, Singapore

## Abstract

**Background and Aims:**

With distinct mechanisms of action, the combination of tropifexor (TXR) and cenicriviroc (CVC) may provide an effective treatment for NASH. This randomized, multicenter, double-blind, phase 2b study assessed the safety and efficacy of TXR and CVC combination, compared with respective monotherapies.

**Approach and Results:**

Patients (N = 193) were randomized 1:1:1:1 to once-daily TXR 140 μg (TXR_140_), CVC 150 mg (CVC), TXR 140 μg + CVC 150 mg (TXR_140_ + CVC), or TXR 90 μg + CVC 150 mg (TXR_90_ + CVC) for 48 weeks. The primary and secondary end points were safety and histological improvement, respectively. Rates of adverse events (AEs) were similar across treatment groups. Pruritus was the most frequently experienced AE, with highest incidence in the TXR_140_ group (40.0%). In TXR and combination groups, alanine aminotransferase (ALT) decreased from baseline to 48 weeks (geometric mean change: −21%, TXR_140_; −16%, TXR_140_ + CVC; −13%, TXR_90_ + CVC; and +17%, CVC). Reductions in body weight observed at week 24 (mean changes from baseline: TXR_140_, −2.5 kg; TXR_140_ + CVC, −1.7 kg; TXR_90_ + CVC, −1.0 kg; and CVC, −0.1 kg) were sustained to week 48. At least 1-point improvement in fibrosis stage/steatohepatitis resolution without worsening of fibrosis was observed in 32.3%/25.8%, 31.6%/15.8%, 29.7%/13.5%, and 32.5%/22.5% of patients in the TXR_140_, CVC, TXR_140_ + CVC, and TXR_90_ + CVC groups, respectively.

**Conclusions:**

The safety profile of TXR + CVC combination was similar to respective monotherapies, with no new signals. TXR monotherapy showed sustained ALT and body weight decreases. No substantial incremental efficacy was observed with TXR + CVC combination on ALT, body weight, or in histological end points compared with monotherapy.

## Graphical abstract

**Figure:**
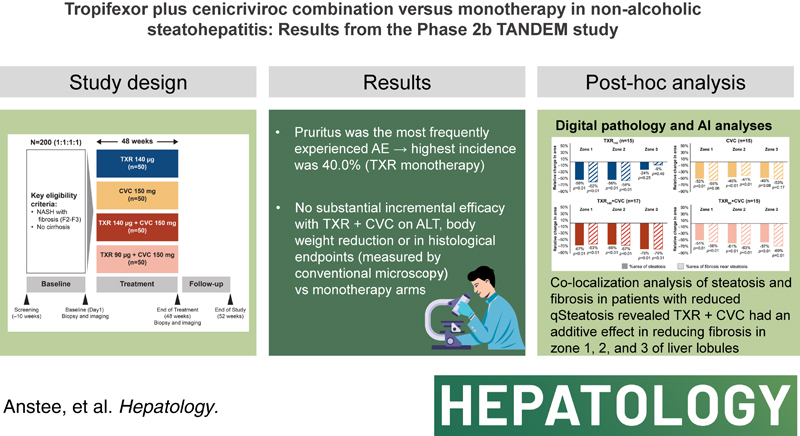

## INTRODUCTION

NASH is a progressive form of NAFLD, one of the most common chronic liver diseases worldwide.^1,2^ It is a chronic inflammatory condition accompanied by hepatocyte damage and varying degrees of fibrosis, which may progress to cirrhosis.^3,4^ Pathogenic mechanisms of NASH are complex and may involve insulin resistance, accumulation of lipids, lipotoxicity, oxidative stress and injury, and gut microbiota disruption.^5–7^

Therapies targeting different pathogenic pathways in NASH are under investigation including nuclear receptor agonists like farnesoid X receptor (FXR), antioxidants, anti-inflammatory agents, antifibrotic agents, and modulators of the TNF-α pathway, among others.^3,8,9^

There are no approved therapies globally for NASH, with many of the investigational agents studied to date either failing to meet histological end points or demonstrating limited efficacy.^8,10^ Given the complex pathophysiological processes that underpin NASH, it is hypothesized that combination therapy targeting multiple distinct mechanisms may effectively control the disease.^3^

Tropifexor (TXR), a potent nonbile acid FXR agonist, has been shown to be highly efficacious in animal models^11,12^ and well tolerated at single doses up to 3000 μg in healthy volunteers with a pharmacokinetic (PK) profile suitable for once-daily dosing.^13^ In the phase 2a/b FLIGHT-FXR study, sustained decreases in alanine aminotransferase (ALT) and hepatic fat fraction (HFF) were observed in patients treated with TXR versus placebo.^14^ Further, the therapeutic effect of FXR agonism in NASH has been demonstrated in clinical trials, with the bile acid derivative obeticholic acid resulting in fibrosis reduction and improvement in the key features of steatohepatitis.^15,16^

Cenicriviroc (CVC), a potent inhibitor of C-C chemokine receptor types 2/5 (CCR2/5), has demonstrated antifibrotic and anti-inflammatory properties in animal models.^17–19^ In the phase 2b CENTAUR study in patients with NASH, 1-year treatment with CVC resulted in an improvement in fibrosis without worsening of steatohepatitis, reduction of biomarkers of inflammation, and comparable safety and tolerability versus placebo.^20^ With distinct and complimentary mechanisms of action, the combination of TXR and CVC might improve efficacy while maintaining safety, thereby leading to potential additive effects. In a preclinical model of diet-induced NASH (streptozotocin administered to neonatal mice, followed by a high-fat diet), treatment with the combination of TXR and CVC showed synergistic reduction in inflammation and ballooning versus monotherapy.^21^ In a drug-drug interaction study in healthy volunteers, this combination exhibited acceptable safety and tolerability versus the respective monotherapy; however, the coadministration of CVC reduced TXR peak drug concentration (C_max_) and area under the concentration-time curve (AUC) by 35% at steady state, while TXR did not influence CVC PKs.^22^ Therefore, it is important to consider exposure differences when comparing the performance of TXR alone and in combination with CVC.

The purpose of this study was to assess the safety and efficacy of the TXR and CVC combination in patients with noncirrhotic NASH compared with respective monotherapies.

## METHODS

### Study design and treatments

TANDEM (NCT03517540) was a 48-week, phase 2b randomized, multicenter, double-blind study conducted between September 2018 (first patient, first visit) and October 2020 (last patient, last visit) in 65 centers across 17 countries. The study design of TANDEM has been reported in detail^23^ and is also illustrated in Supplemental Figure S1, http://links.lww.com/HEP/H829. All eligible patients were randomized to 1 of 4 treatment arms [TXR 140 μg once daily (qd; TXR_140_); CVC 150 mg qd (CVC); TXR 140 μg + CVC 150 mg qd (TXR_140_ + CVC); and TXR 90 μg + CVC 150 mg qd (TXR_90_+CVC)] at a ratio of 1:1:1:1 in a blinded, unbiased manner using Interactive Response Technology. Randomization was stratified by participation in MRI-proton density fat fraction (MRI-PDFF) assessment, which allowed for a balanced number of patients who underwent MRI-PDFF in each treatment arm. Investigators, persons performing the assessments, and the Novartis clinical trial team were blinded to the identity of study treatments from the time of randomization until final database lock.

The original study treatment duration was 48 weeks. A protocol amendment allowed treatment to continue for up to ~8 additional weeks for patients who were unable to attend the study site for the scheduled week 48 end-of-treatment (EOT) assessments, including EOT liver biopsy, due to COVID-19 pandemic-related restrictions, which included temporary closures of clinics. The study protocol and all amendments were reviewed by an Independent Ethics Committee or Institutional Review Board for each center, and the study was conducted according to the ICH E6 Guidelines for Good Clinical Practice that have their origin in the Declaration of Helsinki. All patients provided written informed consent before any study-specific procedures were performed.

### Study population

Male and female patients aged ≥18 years (at the time of the screening visit) weighing between 50 and 200 kg were eligible to participate in the study. Other key inclusion criteria were (1) an adequate liver biopsy sample for evaluation by a central reader, (2) presence of NASH as demonstrated by histologic evidence, and (3) presence of fibrosis stages F2/F3 as demonstrated on a liver biopsy with evaluation by a central reader during the screening period (as per NASH clinical research network [CRN] staging criteria).^24^ Alternatively, a historical biopsy was used if it was performed within 6 months before screening and evaluable by a central reader.

Key exclusion criteria included (1) current or history of significant alcohol consumption for a period of >3 consecutive months within 1-year before screening (>20 g/d in females and >30 g/d in males), (2) uncontrolled diabetes [glycosylated hemoglobin (HbA1c) ≥9% at screening], (3) clinical evidence of hepatic decompensation or severe liver impairment, (4) previous diagnosis of other forms of chronic liver disease or a history of autoimmune liver disease, and (5) women of childbearing potential or pregnant/lactating women.

### Study end point

The primary objective/end point of TANDEM was to evaluate the safety and tolerability of TXR plus CVC in patients with NASH and fibrosis (stages F2/F3) by monitoring adverse events (AEs), vital signs, and laboratory values during 48 weeks of treatment as compared with TXR and CVC monotherapy. This primary objective was chosen as this was the first study to investigate combined FXR agonism with CCR2/5 antagonism in patients with NASH. The secondary end points of this study were to evaluate the proportion of patients who had at least a 1-point improvement in fibrosis stage (NASH CRN) without worsening of steatohepatitis and the proportion of patients with resolution of steatohepatitis without worsening of fibrosis, after 48 weeks of treatment. Exploratory end points included were reported^23^ and provided in the Supplemental Information, http://links.lww.com/HEP/H829.

### Study assessments

Safety assessments were performed to assess the occurrence of AEs, serious AEs (SAEs), AEs leading to study discontinuation or dose reduction, AEs of special interest (AESIs), changes in vital signs, and changes in laboratory data. AEs, SAEs, and vital signs were assessed at screening; baseline; weeks 2, 4, 6, 8, 12, 16, 24, 32, 40, and 48; and post-treatment follow-up at week 52.

To assess treatment-induced changes, paired liver biopsies (baseline and EOT) were reviewed by a central pathologist (blinded to visits and treatment) to score fibrosis staging and grading of steatohepatitis features (steatosis, lobular inflammation, and hepatocyte ballooning). Real-time readings of liver biopsies were also performed at baseline to assess eligibility for entry into the study and after treatment completion. See Supplemental Information, http://links.lww.com/HEP/H829 for further details on liver biopsy readings.

The NAFLD activity score (NAS) was calculated according to the NASH CRN criteria,^24^ which included steatosis (0–3), lobular inflammation (0–3), and hepatocyte ballooning (0–2), giving a range of 0–8 for the NAS.

MRI-PDFF was performed optionally in a subset of patients to quantify HFF at screening. All MRI scans were performed locally and sent to the central MRI laboratory for evaluation. Anthropometric assessments included height and body weight.

Blood samples and fasting blood samples were collected to assess liver biochemistry, which included ALT, aspartate aminotransferase (AST), gamma-glutamyl transferase (GGT), and lipid panels (ie, total cholesterol, HDL cholesterol, LDL cholesterol, triglycerides, free glycerol, and free fatty acids).

A number of noninvasive tests were performed to assess liver damage and function. See Supplemental Information, http://links.lww.com/HEP/H829 for further details.

Patient-reported outcomes included the visual analog scale (VAS) for itch, VAS for sleep disturbance due to itch, Patient Global Impression of change (PGIC; end of study [EOS] only), and Patient Global Impression of severity (PGIS).

Predose and postdose PK plasma samples were collected at specified time intervals over the study duration (weeks 4, 8, 12, 24, and 48). See Supplemental Information, http://links.lww.com/HEP/H829 for further details.

### Statistical analysis

Summary tables are presented by treatment group and analysis visit (as applicable) using descriptive statistics. Continuous variables are summarized by arithmetic mean and SD unless otherwise stated. The number and percentage of patients in each category are presented for categorical variables for each treatment group and all patients (total).

There were no prespecified hypotheses and statistical models in this study. The primary safety variables were analyzed descriptively using a summary table of absolute and relative frequencies, overall and by preferred term (for the occurrence of AEs, SAEs, and AEs leading to study discontinuation or dose reduction), and using a summary table of absolute and relative frequencies, overall and by type of AEs (for AESIs). Only treatment-emergent adverse events were considered for the analysis.

Due to the nature of the primary objective, the assessment was made based on the whole safety profile and not on quantitatively formulated hypotheses for distinct parameters. Therefore, the sample size was based on the feasibility with respect to expected speed of enrollment and duration of the study.

For power considerations, events with a true incidence of 30% and above are likely to be observed (almost 100% probability) in a group of 50 patients (size of each treatment group). Events with true incidences below 10% down to 3% are still very likely to be observed, while events with <50% probability are observed only if the true incidence is less than about 2.5%.

For the secondary objectives, the difference in the proportion of patients on the different TXR plus CVC regimens who achieved at least a 1-point improvement in fibrosis stage and/or resolution of steatohepatitis at week 48 was compared with TXR and CVC monotherapy patients. Treatment differences between TXR + CVC combination therapy and monotherapy with TXR or CVC were evaluated using a Cochran-Mantel-Haenszel test that controlled for baseline fibrosis stage (F2/F3).

For the PK analysis, dose-response and exposure-response relationships for TXR, CVC, and TXR + CVC combination therapy with key selected safety (eg, ALT and AST) and efficacy end points including biomarkers (eg, FGF19 and GGT) were explored.

### Post hoc digital pathology and artificial intelligence analyses of treatment-induced changes in steatosis, hepatocyte ballooning, and liver fibrosis

Unstained, formalin-fixed sections from paired liver biopsies (baseline and EOT) were examined using second harmonic generation/two-photon excitation fluorescence (SHG/TPEF) microscopy with computer-assisted analyses. The liver sections were de-paraffinized, and tissue scanning was performed on Genesis200 (a fully automated, stain-free multiphoton fluorescence imaging microscope) and analyzed using artificial intelligence (AI)-based algorithms (HistoIndex Pte. Ltd) to quantitatively assess qFibrosis, qSteatosis, and qBallooning, as described.^25–27^ Additional analyses were also performed including (1) quantitation of fibrosis dynamics in different zones of liver lobules from baseline to week 48, (2) colocalization analysis to assess the relationship between treatment-induced changes of fibrosis in relation steatosis changes, and (3) colocalization analysis to assess the relationship between treatment-induced changes of fibrosis and hepatocyte ballooning, as described.^27^

To compare steatosis and liver fibrosis changes from baseline and after 48 weeks of treatment, as assessed by NASH CRN scoring and by AI digital quantitation (qSteatosis and qFibrosis), patients in the 4 treatment arms were categorized as Progressor, No Change, or Regressor (*P*/N/R analysis). The qSteatosis and qFibrosis results were expressed by both categorial steatosis grade (qS0 to qS3) and fibrosis stage (qF0 to qF4), respectively, as well as a continuous value. For steatosis, progression was defined as an increase of ≥1 grade from baseline to week 48 or an increase of ≥1 SEM (for qSteatosis as a continuous value); regression was defined as a decrease of ≥1 grade or a decrease of ≥1 SEM (for qSteatosis as a continuous value). For fibrosis, progression was defined as an increase of ≥1 stage or an increase of ≥1 SEM (for qFibrosis as a continuous value); regression was defined as a decrease of ≥1 stage or ≥1 SEM (for qFibrosis as a continuous value). See Supplemental Information, http://links.lww.com/HEP/H829 for further details.

## RESULTS

### Patient disposition and baseline demographics

**FIGURE 1:**
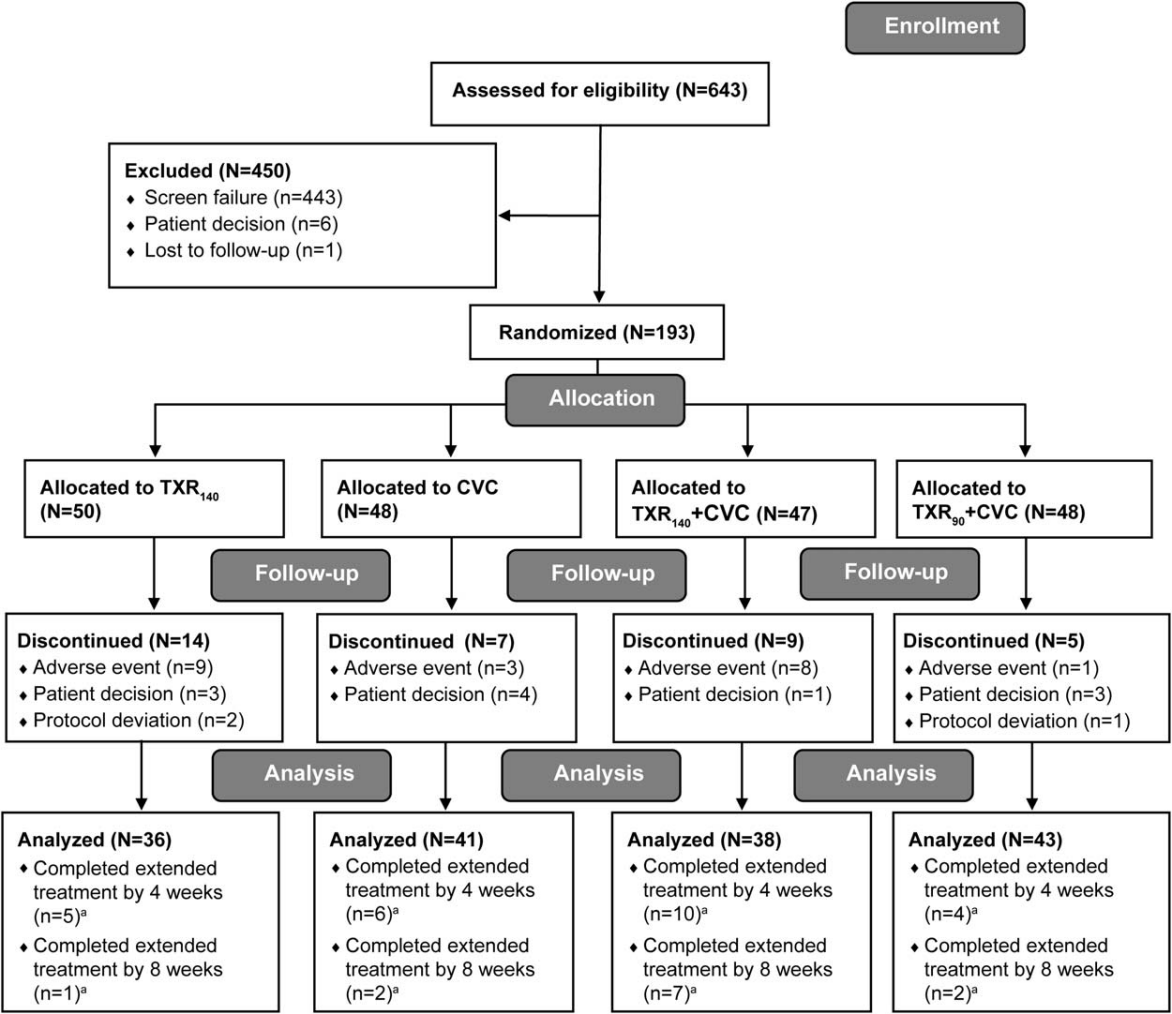
CONSORT flow diagram of participant disposition by treatment group. ^a^If a patient completed the 8-week extension, the patient was also counted as having completed the 4-week extension. Abbreviations: CVC, cenicriviroc; N, number of patients in group; n, number of patients with outcome; TXR_90_, tropifexor 90 µg; TXR_140_, tropifexor 140 µg.

Overall, 643 patients were screened for study inclusion, with 193 patients (30.0%) meeting the entry criteria for randomization. Of the 193 patients who were randomized to study treatment, 158 patients (81.9%) completed the study (Figure 1).

Baseline patient demographics and characteristics are displayed in Table 1. Across treatment groups, mean ages of patients ranged from 54 to 55 years, most patients were female (60.0%–64.6%), except in the TXR_90_ + CVC group (47.9% were female), and most were Caucasian (82.0%–91.7%). Across treatment groups, 82.4% of patients were reported to have diabetes and 55.4% were taking concomitant lipid-reducing medication.

**TABLE 1:** Demographics and baseline characteristics

Demographics and baseline characteristics	TXR_140_ N = 50	CVC N = 48	TXR_140_ + CVC N = 47	TXR_90_ + CVC N = 48
Age (y), mean (± SD)	54.8 ± 13.4	53.7 ± 11.8	54.7 ± 12.7	54.9 ± 12.3
Male, n (%)	20 (40.0)	17 (35.4)	18 (38.3)	25 (52.1)
Race, n (%)				
Caucasian	41 (82.0)	44 (91.7)	40 (85.1)	43 (89.6)
Asian	7 (14.0)	4 (8.3)	5 (10.6)	5 (10.4)
Black	1 (2.0)	0	2 (4.3)	0 (0.0)
Unknown	1 (2.0)	0	0	0
BMI (kg/m^2^), mean (± SD)	33.7 ± 6.6	35.7 ± 8.4	34.7 ± 6.9	34.3 ± 7.3
Diabetes, n (%)	39 (78.0)	41 (85.4)	39 (83.0)	40 (83.3)
Lipid-lowering medication, n (%)	22 (44.0)	23 (47.9)	33 (70.2)	29 (60.4)
AST, mean (±SD)	38.6 ± 18.0	35.1 ± 13.0	42.1 ± 25.2	41.4 ± 25.8
ALT, mean (±SD)	49.1 ± 25.3	40.9 ± 15.7	51.1 ± 28.6	51.8 ± 33.0
ELF, mean (±SD)	9.6 ± 0.8	9.5 ± 1.0	9.4 ± 0.7	9.6 ± 0.8
HOMA-IR, mean (±SD)	8.4 ± 7.6	7.0 ± 4.8	7.1 ± 6.2	10.5 ± 14.5
FIB-4, mean (±SD)	1.3 ± 0.7	1.3 ± 0.6	1.4 ± 0.9	1.3 ± 0.8
Histological characteristics				
Fibrosis (NASH CRN), real-time biopsy^a^, n (%)				
Stage 1	0	0	0	0
Stage 2	17 (34.0)	10 (20.8)	10 (21.3)	17 (35.4)
Stage 3	32 (64.0)	38 (79.2)	37 (78.7)	30 (62.5)
Stage 4	1 (2.0)	0	0	1 (2.1)
NASH diagnosis by CRN, n (%)	50 (100.0)	48 (100.0)	47 (100.0)	48 (100.0)
NAS total score (mean ± SD)	5.2 ± 0.93	5.4 ± 0.82	5.4 ± 1.06	5.2 ± 1.02
Steatosis, n (%)				
1 (5%–33%)	42 (84.0)	38 (79.2)	39 (83.0)	41 (85.4)
2 (34%–66%)	8 (16.0)	10 (20.8)	8 (17.0)	7 (14.6)
Lobular inflammation, n (%)				
1 (< 2 foci/*field)	3 (6.0)	2 (4.2)	3 (6.4)	4 (8.3)
2 (2–4 foci/*field)	24 (48.0)	24 (50.0)	18 (38.3)	25 (52.1)
3 (>4 foci/*field)	23 (46.0)	22 (45.8)	26 (55.3)	19 (39.6)
Hepatocyte ballooning, n (%)				
1 (mild, few)	17 (34.0)	11 (22.9)	12 (25.5)	13 (27.1)
2 (moderate, many)	33 (66.0)	37 (77.1)	35 (74.5)	35 (72.9)

a Real-time readings of liver biopsies were performed at baseline to assess eligibility for entry into the study.

Abbreviations: ALT, alanine aminotransferase; AST, aspartate aminotransferase; BMI, body mass index; CRN, clinical research network; CVC, cenicriviroc; ELF, enhanced liver fibrosis; FIB-4, fibrosis-4; HOMA-IR, homeostatic model assessment for insulin resistance; N, number of patients in group; n, number of patients with outcome; NAS, NAFLD activity score; TXR_90_, tropifexor 90 µg; TXR_140_, tropifexor 140 µg.

Based on baseline liver biopsies and NASH CRN histological scoring, F3 fibrosis was present in 64.0%, 79.2%, 78.7%, and 62.5% of patients in the TXR_140_, CVC, TXR_140_ + CVC, and TXR_90_ + CVC groups, respectively. All patients had NAS: steatosis (1 or 2), with lobular inflammation (1, 2, or 3), and hepatocyte ballooning (1 or 2). The mean NAS total score was 5.2 in the TXR_140_ and TXR_90_ + CVC groups and 5.4 in the CVC and TXR_140_ + CVC groups.

### Safety

Rates of AEs were similar across treatment groups. Overall, 85.5% of patients experienced at least 1 AE (Table 2).

**TABLE 2:** Occurrence of AEs during the study and TEAEs with ≥10% incidence, by preferred term

Events, n (%)	TXR_140_ N = 50	CVC N = 48	TXR_140_ + CVC N = 47	TXR_90_ + CVC N = 48	Total N = 193
Patients with at least 1 AE	42 (84.0)	41 (85.4)	40 (85.1)	42 (87.5)	165 (85.5)
Patients with at least 1 SAE	5 (10.0)	3 (6.3)	4 (8.5)	10 (20.8)	22 (11.4)
AE as reason for discontinuation	9 (18.0)	3 (6.3)	8 (17.0)	1 (2.1)	21 (10.9)
TEAEs (incidence of ≥10% in any treatment group), n (%)					
Pruritus	20 (40.0)	10 (20.8)	15 (31.9)	10 (20.8)	55 (28.5)
Nausea	2 (4.0)	6 (12.5)	7 (14.9)	6 (12.5)	21 (10.9)
Fatigue	7 (14.0)	4 (8.3)	5 (10.6)	4 (8.3)	20 (10.4)
Arthralgia	6 (12.0)	3 (6.3)	6 (12.8)	1 (2.1)	16 (8.3)
Constipation	5 (10.0)	2 (4.2)	6 (12.8)	3 (6.3)	16 (8.3)
Urinary tract infection	7 (14.0)	3 (6.3)	2 (4.3)	4 (8.3)	16 (8.3)
Abdominal pain	5 (10.0)	3 (6.3)	5 (10.6)	2 (4.2)	15 (7.8)
Upper respiratory tract infection	3 (6.0)	2 (4.2)	5 (10.6)	5 (10.4)	15 (7.8)
Asthenia	4 (8.0)	2 (4.2)	5 (10.6)	3 (6.3)	14 (7.3)
Back pain	1 (2.0)	3 (6.3)	5 (10.6)	4 (8.3)	13 (6.7)
Diarrhea	2 (4.0)	7 (14.6)	4 (8.5)	0	13 (6.7)
Abdominal pain upper	3 (6.0)	2 (4.2)	5 (10.6)	2 (4.2)	12 (6.2)

*Note:* A patient with multiple occurrences of an AE is counted only once.

Abbreviations: AE, adverse event; CVC, cenicriviroc; N, number of participants in group; n, number of participants with outcome; SAE, serious AE; TEAE, treatment-emergent AE; TXR_90_, tropifexor 90 µg; TXR_140_, tropifexor 140 µg.

The proportion of patients who experienced pruritus was the highest in the TXR_140_ group (40.0%) and was lower in the TXR_140_ + CVC group (31.9%). Similar patterns were noted for fatigue and urinary tract infection.

Overall, 21 of 193 patients (10.9%) discontinued from study treatment due to an AE, with most discontinuations occurring in the TXR_140_ group (9/50, 18.0%) and in the TXR_140_ + CVC group (8/47, 17.0%).

The AEs that attributed to the most discontinuations from study treatment were pruritus [TXR_140_ (4/50, 8.0%) and TXR_140_ + CVC (2/47, 4.3%)] and flatulence [TXR_140_ + CVC (2/47, 4.3%)]; all other AEs leading to discontinuation were single occurrences. SAEs were reported in 22 of 193 patients (11.4%) overall, and the incidence was the highest in the TXR_90_ + CVC group. Most SAEs were single occurrences, except for 2 patients who experienced SAEs due to COVID-19 (TXR_90_ + CVC group). Of the 22 patients with SAEs, only 1 patient had an SAE that was attributed by the investigator to study treatment (spondylitis, TXR_140_ group). A cerebrovascular accident was reported in 1 participant each in the TXR_140_ + CVC and TXR_90_ + CVC groups.

### Liver tests, hepatic fat fraction, and fibrosis markers

**FIGURE 2:**
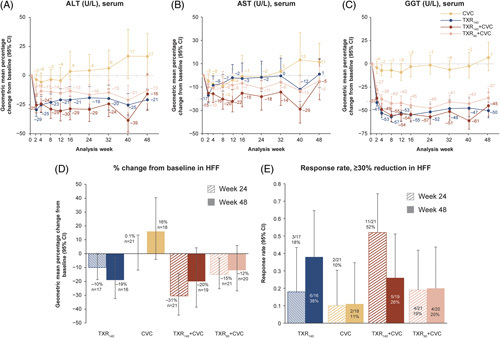
Geometric mean percentage change from baseline (95% CI) up to week 48 in all groups and change in hepatic fat fraction at weeks 24 and 48. (A) Geometric mean percentage change (95% CI) in ALT; (B) geometric mean percentage change (95% CI) in AST; (C) geometric mean percentage change (95% CI) in GGT; (D) geometric mean percentage change from baseline in hepatic fat fraction; and (E) patients with at least a 30% reduction in hepatic fat fraction. Abbreviations: ALT, alanine aminotransferase; AST, aspartate aminotransferase; CVC, cenicriviroc; GGT, gamma-glutamyl transferase; HFF, hepatic fat fraction; n, number of patients in group; TXR_90_, tropifexor 90 µg; TXR_140_, tropifexor 140 µg.

In the TXR and combination groups, a decrease in ALT (Figure 2A), AST (Figure 2B), and GGT (Figure 2C) from baseline was noted during the 48-week period. Approximately 50% of patients underwent MRI-PDFF. At both weeks 24 and 48, reductions in HFF were observed in all TXR-containing groups, with the highest percentage reduction observed with TXR_140_ + CVC combination therapy (Figure 2D). Reductions of at least a 30% in HFF at weeks 24 and 48, as measured by PDFF, were observed more frequently in all TXR-containing groups compared with CVC monotherapy group (Figure 2E). At weeks 24 and 48, the number of patients with at least a 30% reduction in HFF was the highest in the TXR_140_ + CVC (11/21, 52%) and TXR_140_ (6/16, 38%) groups (Figure 2E).

There were no consistent changes from baseline to week 48 for liver stiffness (via Fibroscan), enhanced liver fibrosis scores, fibrosis biomarker test scores, NAFLD fibrosis scores, or magnetic resonance elastography-derived liver stiffness from baseline to week 48 with any TXR-containing dose. At week 48, although there was a slight reduction in post-treatment mean fibrosis-4 (FIB-4) from baseline (change from baseline: −0.02) in the TXR_140_ + CVC group, when compared with the change from baseline in the monotherapy and TXR_90_ + CVC groups, the change was not considered meaningful. Further details of fibrosis markers and other biomarker results are given in Supplemental Figures S2, S3, http://links.lww.com/HEP/H829).

### Liver histology

**FIGURE 3:**
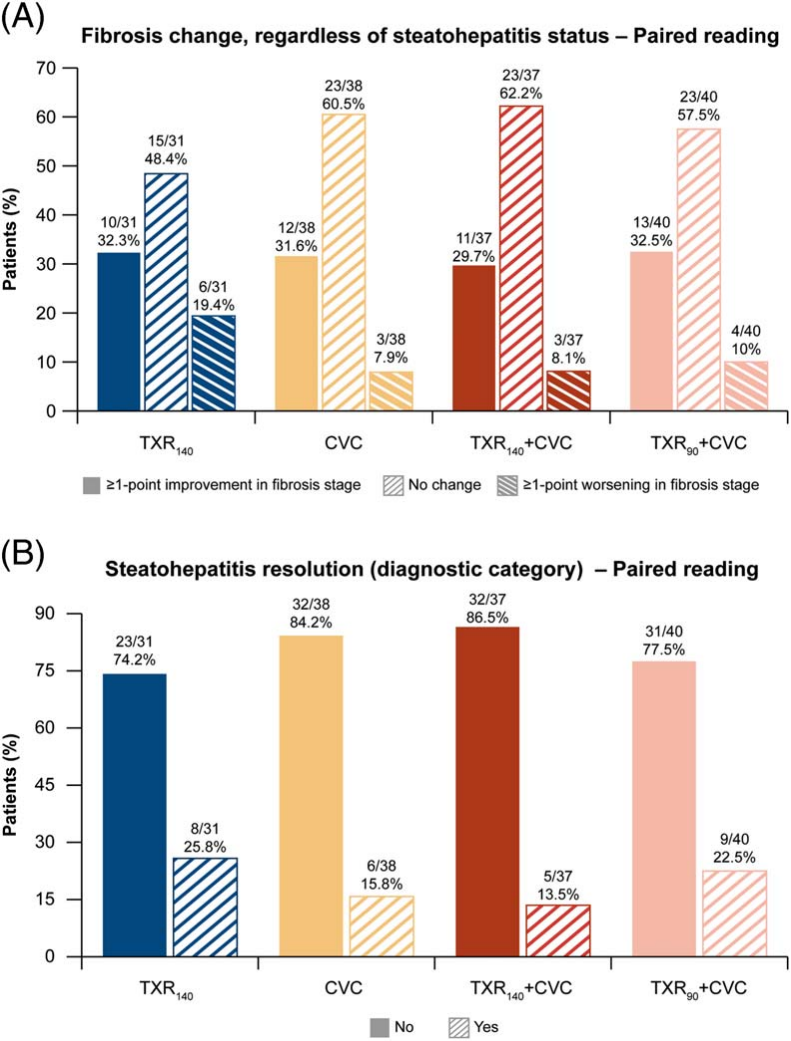
Participants’ histological response at week 48 in all groups based on NASH CRN staging. (A) Proportion of patients with change in fibrosis stage and (B) proportion of patients with steatohepatitis resolution. Abbreviations: CRN, clinical research network; CVC, cenicriviroc; TXR_90_, tropifexor 90 g; TXR_140_, tropifexor 140 µg.

At least a 1-point improvement in fibrosis stage was observed in 32.3% (10/31), 31.6% (12/38), 29.7% (11/37), and 32.5% (13/40) of patients in the TXR_140_, CVC, TXR_140_ + CVC, and TXR_90_ + CVC groups, respectively (Figure 3A). Steatohepatitis resolution without worsening of fibrosis was observed in 25.8% (8/31), 15.8% (6/38), 13.5% (5/37), and 22.5% (9/40) of patients in the TXR_140_, CVC, TXR_140_ + CVC, and TXR_90_ + CVC groups, respectively (Figure 3B). There was no evidence that patients who received combination therapy demonstrated a higher likelihood of at least a 1-stage improvement in fibrosis (NASH CRN staging) or achieving resolution of steatohepatitis after 48 weeks of treatment compared with patients who received monotherapy treatment, based on paired biopsy readings. The OR and 95% CI were similar in the TXR_90_ + CVC group compared with CVC (1.21, 95% CI, 0.41, 3.61) and lowest in the TXR_140_ + CVC group compared with TXR_140_ (0.8, 95% CI, 0.25, 2.63). Similar results were reported based on real-time biopsies (see Supplemental Figure S4, http://links.lww.com/HEP/H829) and for the histologic end point based on the Food and Drug Administration (FDA)/European Medicines Agency (EMA) definition of resolution of steatohepatitis without worsening of fibrosis (NASH CRN staging) (Supplemental Table S1, http://links.lww.com/HEP/H829).

The proportion of patients with paired biopsy results at week 48 with at least 2-point improvement in fibrosis (NASH CRN staging) regardless of steatohepatitis status or without worsening of steatohepatitis at 48 weeks of treatment was considered not to be clinically meaningful (Supplemental Table S2, http://links.lww.com/HEP/H829). Changes in individual histological features of NASH are shown in Supplemental Table S3, http://links.lww.com/HEP/H829.

At week 48, no consistent changes in liver collagen morphometry were observed in combination treatment groups, with the highest response in the TXR_140_ + CVC group compared with TXR_140_ monotherapy group [0.7 (adjusted mean difference vs. TXR_140_)].

### Digital quantitation and AI analyses of treatment-induced changes in steatosis, ballooning, and liver fibrosis

**FIGURE 4:**
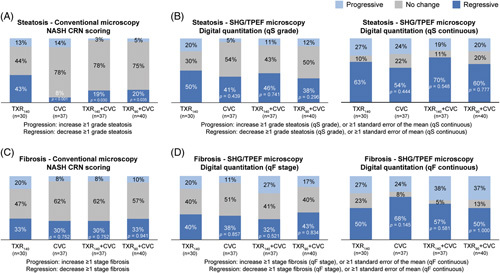
Changes in steatosis and liver fibrosis as assessed by NASH CRN scoring and by digital quantification from baseline to week 48. (A) *P*/N/R analysis of steatosis changes based on the NASH CRN scoring; (B) *P*/N/R analysis of steatosis changes based on the digital quantitation and expressed as qSteatosis grade or qSteatosis as a continuous value; (C) *P*/N/R analysis of fibrosis changes based on the NASH CRN scoring; and (D) *P*/N/R analysis of fibrosis changes based on the digital quantitation and expressed as qFibrosis stage or qFibrosis as a continuous value. *p*-values obtained by comparing each treatment arm versus TXR 140 µg monotherapy using a chi-squared test. Abbreviations: CRN, Clinical Research Network; CVC, cenicriviroc; n, number of patients per group; *P*/N/R, Progressive/No-change/Regressive; qF, qFibrosis; qS, qSteatosis; SHG/TPEF, second harmonic generation/two-photon excitation fluorescence microscopy; TXR_90_, tropifexor 90 µg; TXR_140_, tropifexor 140 µg.

Paired liver biopsies from 144 patients (TXR_140_, N = 30; CVC, N = 37; TXR_140_ + CVC, N = 37; TXR_90_ + CVC, N = 40) were analyzed. *P*/N/R analyses revealed that TXR_140_ alone or in combination with CVC had a greater effect in reducing steatosis than CVC monotherapy. The TXR antisteatotic effect observed in the TXR_140_ group was significantly greater than the other 3 treatment groups based on the NASH CRN scoring (Figure 4A), as well as numerically higher with the digital quantitation of qSteatosis changes (Figure 4B). No significant difference was present among groups when assessing liver fibrosis changes from baseline to week 48 either with the NASH CRN scoring or with qFibrosis (stage and continuous value) digital quantitation (Figure 4 C, D).

**FIGURE 5:**
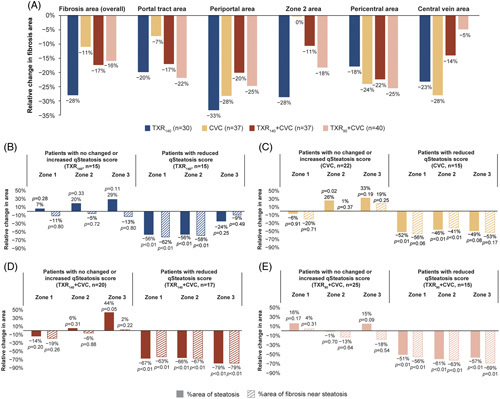
Treatment-induced fibrosis changes in different zones of liver lobules and colocalization analysis of steatosis and fibrosis changes from baseline to week 48. (A) Digital quantification of fibrosis dynamics as a percentage change of fibrosis area in different zones of the liver lobule. The periportal and pericentral areas are set at 100 µm from the portal tract and the central vein, respectively, and the region in between is the Zone 2 area. *P >* 0.05 each treatment group versus TXR_140_ treatment group (Chi-squared test); (B–E) Colocalization analysis of steatosis and fibrosis in zone 1, 2, and 3 of liver lobules. Patients were divided into 2 subgroups: those with “unchanged” or “increased” qSteatosis and those with “reduced” qSteatosis. Wilcoxon rank test was used for *p*-values comparing baseline to week 48 changes. Abbreviations: CVC, cenicriviroc; n, number of patients per group; TXR_90_, tropifexor 90 µg; TXR_140_, tropifexor 140 µg.

In-depth analyses of fibrosis changes in different zones of liver lobule demonstrated that TXR_140_ achieved greater fibrosis reduction overall and in the periportal area (−33%) and in zone 2 (−28%) (Figure 5A). The antifibrotic effect of CVC monotherapy was seen mainly in the periportal (28%), pericentral (24%), and central vein (28%) areas (Figure 5A). In the colocalization analyses, significant fibrosis reduction was observed only in association with steatosis reduction in all treatment groups. The effect of TXR_140_ monotherapy was seen mainly in zone 1 and zone 2 (Figure 5B); the effect of CVC monotherapy appeared uniform in all 3 zones of liver lobule (Figure 5C), while both TXR_140_ + CVC and TXR_90_ + CVC treatment groups showed an additive effect, achieving the greatest fibrosis reduction near steatosis in all 3 zones of the liver lobule, with the TXR_140_ + CVC group showing the greatest treatment-induced changes (Figure 5D, E).

There were no notable changes in the number of ballooned hepatocytes from baseline to week 48 with any of the 4 treatment regimens, irrespective of whether patients had a high or low number of ballooned hepatocytes. (Supplemental Figure S5A, http://links.lww.com/HEP/H829). Treatment-induced changes of fibrosis in relation to hepatocyte ballooning, in the 4 treatment arms, were evaluated by simultaneous measurement of fibrosis and hepatocyte ballooning in colocalization analyses. A marked increase of qBallooning area (as observed in the TXR_140_ + CVC group) was associated with a relative increase (%) in fibrosis area near ballooned hepatocytes, while improvement in qBallooning area was associated with reduction of the nearby collagen fibers. In the subset of patients who had improved qBallooning grade, the TXR_140_ group showed the highest improvement in qBallooning area (67% reduction) and in nearby fibrosis (62% reduction), compared with the other treatment groups. (Supplemental Figure S5B, C, http://links.lww.com/HEP/H829).

### Lipid parameters, body weight, and HOMA-IR

**FIGURE 6:**
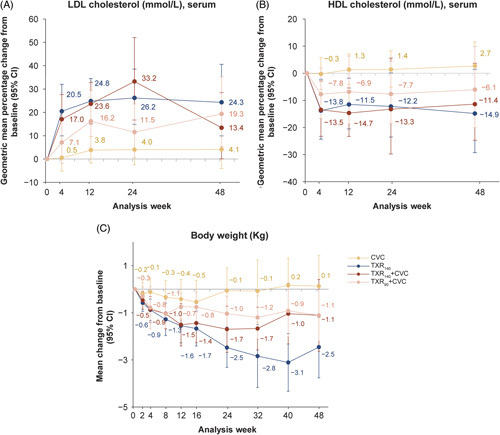
Change in lipid parameters and body weight up to week 48. (A) Geometric mean percentage change (95% CI) in LDL cholesterol; (B) geometric mean percentage change (95% CI) in HDL cholesterol; and (C) mean change from baseline (95% CI) in body weight up to week 48 in all groups. Abbreviations: CVC, cenicriviroc; TXR_90_, tropifexor 90 µg; TXR_140_, tropifexor 140 µg.

Mean LDL cholesterol increased (Figure 6A) and mean HDL cholesterol decreased (Figure 6B) from baseline in the TXR_140_ group and both combination treatments, but there was less change with CVC. In both cases, the treatment effect was apparent by week 4 and near maximum by week 12 with little change thereafter. At week 48, in the TXR_140_ group and combination treatment groups, the mean increase in LDL cholesterol from baseline ranged between 0.43 and 0.56 mmol/L (17–22 mg/dL) and the mean decrease in HDL cholesterol from baseline ranged between 0.07 and 0.20 mmol/L (3 and 8 mg/dL). For the other lipids (total cholesterol, triglycerides, free glycerol, and free fatty acids), there was very little change from baseline or a mild-to-moderate worsening in lipid levels during the study, with little suggestion of a difference between treatment groups (Supplemental Figure S6, http://links.lww.com/HEP/H829). At baseline, the proportion of patients taking lipid-lowering agents was higher in combination treatment arms [70.2% (TXR_140_ + CVC); 60.4% (TXR_90_ + CVC)] compared with monotherapy arms [44.0% (TXR_140_); 47.9% (CVC)]. During the treatment period, none of the patients started a statin as a new medication in any treatment arm.

A reduction in body weight from baseline was evident with TXR-containing groups by week 4. By week 24, the following mean changes from baseline were reported: TXR_140_, −2.5 kg; TXR_140_ + CVC, −1.7 kg; TXR_90_ + CVC, −1.0 kg; and CVC, −0.1 kg. These reduction patterns were sustained until week 48 (Figure 6C).

Median HOMA-IR scores at baseline ranged between 4.60 and 6.63 across treatment groups, with the highest baseline score in the TXR_140_ group. At week 24, HOMA-IR scores improved in the TXR_140_ and CVC groups with a change from baseline of −0.78 and −0.16, respectively, while scores in the combination therapy groups increased (change from baseline: 0.68 and 0.80, respectively). At week 48, the HOMA-IR score decreased in the TXR_140_ group only (change from baseline: −0.49) with no difference from baseline in the CVC group and somewhat lower scores in both combination therapy groups (0.34 and 0.57, respectively; see Supplemental Table S4, http://links.lww.com/HEP/H829).

### Patient-reported outcomes

There were no differences in the VAS scores for itch intensity and sleep disturbance due to itch across treatment groups. For patients who responded to the global PGIS questionnaire, results at weeks 12, 24, and 48 were similar to those at baseline across all treatment groups; most patients rated their symptoms as very mild, mild, or moderate. In patients who responded to the PGIC questionnaire, most in the CVC and TXR_140_ + CVC groups indicated that their symptoms were “about the same” (30/44, 68.2%, and 21/41, 51.2%, respectively).

### Pharmacokinetics

No marked difference was present in the TXR predose mean plasma concentration range for the TXR_140_ dose level (1.56–1.95 ng/mL) when dosed either alone or in combination with CVC (1.33–1.91 ng/mL). Reduced TXR predose concentrations were observed in the TXR_90_ + CVC group (1.04–1.13 ng/mL), broadly in proportion to the reduced dose of TXR (Supplemental Figure S7A, http://links.lww.com/HEP/H829). Postdose mean TXR drug concentration ranges were reduced by ~10% in the TXR_140_ + CVC study arm (1.61–1.98 ng/mL) (Supplemental Figure S7B, http://links.lww.com/HEP/H829) compared with the TXR_140_ group (1.91–2.23 ng/mL).

Plasma concentration ranges of CVC in pre- and postdose PK samples were comparable across each of the study arms (predose concentration range, 179–204 ng/mL for the CVC group, 160–198 ng/mL for the TXR_90_ + CVC group, and 165–229 ng/mL for the TXR_140_ + CVC group). Furthermore, CVC plasma concentrations were consistent throughout the study (postdose concentration range, 181–219 ng/mL for the CVC group, 167–215 ng/mL TXR_90_ + CVC group, and 174–226 ng/mL for the TXR_140_ + CVC group) (Supplemental Figure S7C, D, http://links.lww.com/HEP/H829).

## DISCUSSION

This study was a 48-week, randomized, double-blind, multicenter trial with the primary objective to evaluate the safety and tolerability of TXR + CVC in patients with NASH and fibrosis as compared with TXR and CVC monotherapy. The safety profiles of the combination therapies of TXR_140_ + CVC and TXR_90_ + CVC were similar to those of monotherapy with TXR and CVC, with no additional emergent safety signals compared with those identified and reported in previous monotherapy studies, and no deaths were reported.^14,20^

Overall, pruritus, nausea, and fatigue were the most frequently experienced AEs, with the highest incidence of pruritus observed in the TXR monotherapy group and notably lower with TXR_140_ + CVC combination treatment. The observed reduction of postdose TXR levels (~10%) in the presence of CVC may explain this. A previous drug-drug interaction study^22^ showed reduced systemic TXR exposure of 35% for C_max_ and AUC and most likely reflects the true reduction in TXR exposure for the combination, but not monotherapy arms. This may also have had an impact on the observed efficacy of combination therapy as dosed in this trial (discussed below), with a higher TXR dose of 200 μg potentially resulting in greater efficacy than that observed with TXR at a dose of 140 μg. Most SAEs were single occurrences. The AE most frequently leading to discontinuation from study treatment was pruritus. Other studies investigating FXR agonists^14–16,28,29^ have also noted pruritus as a common AE, indicating that it may be a class effect of agonism by FXR.

There was a decrease in ALT, AST, and GGT in the TXR monotherapy and combination groups from baseline during the 48-week period, while in the CVC monotherapy group, no such reduction was observed. A similar decrease in ALT from baseline with TXR was observed in the FLIGHT-FXR study.^14^ With a number of studies suggesting that a ≥30% relative reduction in HFF measured by MRI-PDFF may be associated with histologic response in NASH trials,^30,31^ it is interesting to note that in our study, this level of reduction in HFF was observed in several of the FXR-containing arms without notable histologic response. At week 24, the number of patients with a ≥30% reduction in HFF measured by MRI-PDFF was highest in the TXR_140_ + CVC group (52.4%); however, at week 48, the highest number of patients with a ≥30% reduction in HFF was seen in the TXR_140_ monotherapy group (37.5%). No meaningful change over the study period was observed in noninvasive markers of liver fibrosis.

Although the observed fibrosis improvement was in the same range as in other placebo-controlled monotherapy studies with CVC or TXR,^14,20^ in our study, neither of the combination therapies (TXR_140_ + CVC or TXR_90_ + CVC) increased the likelihood of improvement of fibrosis or resolution of steatohepatitis, based on the central pathologist’s assessment, when compared with either TXR or CVC monotherapy. In the CENTAUR study, ≥1-stage improvement in fibrosis without worsening of steatohepatitis at 1 year was observed in twice as many patients on CVC (20%) compared with placebo.^20^ Interim results of the phase 3 AURORA study (NCT03028740), designed to evaluate and confirm the efficacy and safety of CVC for the treatment of liver fibrosis in adults with NASH, showed a lack of efficacy.^32^

Although we did not see synergistic efficacy with these therapies when used in combination, this does not exclude such an effect in other combinations. As the effects of CVC on metabolic components of NASH are thought to be limited^33^ and its antifibrotic activity did not perform as expected in the AURORA phase 3 study, a TXR combination with another compound with potent antimetabolic activity may have provided greater synergy and efficacy. In addition, it is worth noting that in our study, most patients had markers of progressive NASH at baseline, with stage 3 fibrosis, a proportion that is higher than in other noncirrhotic trials investigating NASH. When considering more advanced NASH, a trial involving patients with advanced fibrosis or cirrhosis (fibrosis stage 3–4), combination therapy with firsocostat (an acetyl-CoA carboxylase inhibitor), and cilofexor (an FXR agonist) resulted in greater improvements in histology and clinically relevant biomarkers versus either agent alone or placebo, although, as with our trial, the histological end point was not met.^29^

The post hoc digital pathology and AI analyses provided useful mechanistic details in the evaluation of NASH treatment with compounds of different mechanisms of action. TXR alone was shown to have greater impact in reducing steatosis than CVC monotherapy, consistent with TXR effects in reducing liver enzymes and HFF; the antifibrotic activity of TXR monotherapy was seen mainly in zone 1 and zone 2 of the liver lobule, while CVC showed antifibrotic effects uniformly in all 3 zones of the liver lobule. Although no significant difference in liver fibrosis stage was observed between the 4 treatment groups over the study period using conventional microscopy, digital quantitation analysis of fibrosis changes in different zones of the liver lobule revealed that the combination of TXR and CVC had an additive effect, achieving the greatest fibrosis reduction near steatosis in all 3 zones of the liver lobule, compared with each monotherapy group, with the TXR_140_ + CVC group showing the greatest treatment-induced changes. Similarly, the use of AI/machine learning analyses of liver biopsies from the ATLAS trial provided greater details than the conventional microscopy and the NASH CRN scoring in evaluating the effects of another combination therapy.^34^ The effects seen in different liver lobules in our trial using digital pathology and AI analyses may not have been detected using conventional microscopy.

The colocalization analyses assessing treatment-induced changes of fibrosis in relation to steatosis or hepatocyte ballooning, in the 4 treatment arms, confirmed the association that fibrosis regression occurs in cases with reduction of steatosis and hepatocyte ballooning, as described.^27^ Overall, these data illustrate the granularity and the additional information that can be obtained by applying AI digital pathology for quantitative assessment of NASH features and liver fibrosis in general and the advantage of its use in clinical trials, along with the standard diagnostic assessment of liver histology. The clinical relevance of AI digital measurements of the NASH features, especially for liver fibrosis progression or regression, will have to be established in future studies in relation to liver-related clinical outcomes.

The treatment effect on changes in lipid levels related to TXR treatment was apparent by week 4, near maximum by week 12, and with little change thereafter. This was similar to the stabilization of changes in lipid levels after week 12 in the FLIGHT-FXR study.^14^ There were no consistent changes in triglycerides in any treatment group. These effects are in line with those observed with other FXR agonists. There was a sustained reduction in body weight in the TXR-containing groups, although the COVID-19 pandemic-related restrictions introduced mid-study may have attenuated the effects seen with TXR on weight loss. Patient-reported outcomes were similar across all treatment groups.

PK data from this study confirm a similar predose exposure between TXR_140_ monotherapy and TXR_140_ combination therapy and a trend for a higher TXR exposure in samples collected after dose for the TXR_140_ monotherapy group. Study end points that are similar for the TXR_140_ monotherapy and TXR_140_ + CVC combination group (eg, lipids, ALT, and GGT) may be more dependent on C_min_, whereas for the stronger effects seen in the TXR_140_ monotherapy group (eg, pruritus and weight loss), the higher total exposure (AUC and C_max_) may contribute.

Limitations of this study include a small number of patients, limiting the power to address histological change, with the COVID-19 pandemic effect further decreasing effective sample size, and leading to missed visits and central laboratory assessments.

## CONCLUSIONS

TXR monotherapy showed sustained decrease in ALT and body weight, but there was no substantial incremental efficacy with the combination of TXR and CVC either on ALT or body weight reduction nor in histological end points when compared with the monotherapy arms. The TANDEM study demonstrated that the safety profile of this combination therapy (TXR + CVC) was similar to that of each of the monotherapies. There were no new safety signals compared with those already reported in monotherapy studies.

## Supplementary Material
