# Supplementary material for: Tropifexor plus cenicriviroc combination versus monotherapy in nonalcoholic steatohepatitis: Results from the phase 2b TANDEM study
Source: Hepatology. 2023 May 11;78(4):1223–39. doi: 10.1097/HEP.0000000000000439 (PMC10521801; doi:10.1097/HEP.0000000000000439)
Supplement: Supplementary file 1 [file hep-78-1223-s001.docx]

# **Supplementary information**

This appendix has been provided by the authors to give readers additional information about their work.

# **Title**

Tropifexor plus cenicriviroc combination versus monotherapy in non-alcoholic steatohepatitis: Results from the Phase 2b TANDEM study

# **Authors**

Quentin M Anstee, Kathryn J Lucas, Sven Francque, Manal F Abdelmalek, Arun J Sanyal, Vlad Ratziu, Adrian C Gadano, Mary Rinella, Michael Charlton, Rohit Loomba, Edward Mena, Jörn M Schattenberg, Mazen Noureddin, Donald Lazas, George BB Goh, Shiv Kumar Sarin, Yusuf Yilmaz, Miljen Martic, Rowan Stringer, Jossy Kochuparampil, Li Chen, Gerardo Rodriguez-Araujo, Elaine Chng, Nikolai V Naoumov, Clifford Brass, Marcos C Pedrosa

**Table of Contents**

[**Supplementary methods** 3](#_Toc122690591)

[**Exploratory study endpoints** 3](#_Toc122690592)

[**Study assessments** 3](#_Toc122690593)

[**Liver biopsies** 3](#_Toc122690594)

[**Non-invasive tests** 4](#_Toc122690595)

[**PK analysis** 4](#_Toc122690596)

[**Post hoc digital pathology and artificial intelligence (AI) analyses of treatment-induced changes in steatosis, hepatocyte ballooning, and liver fibrosis** 4](#_Toc122690597)

[**Supplementary figures** 6](#_Toc122690598)

[**Supplementary Figure 1. Study design** 6](#_Toc122690599)

[**Supplementary Figure 2. Box plots for fibrosis markers. (A) Fibroscan; (B) MRE-liver stiffness; (C) ELF; (D) NAFLD fibrosis score; (E) Fibrosis biomarker test score** 7](#_Toc122690600)

[**Supplementary Figure 3. Mean change from baseline in (A) FIB-4 and (B) High-sensitivity C-reactive protein** 8](#_Toc122690601)

[**Supplementary Figure 4. Patients with histological responses at Week 48 in all groups based on NASH CRN staging: real-time reading. (A) Fibrosis improvement (≥1 point, NASH CRN staging); (B) Steatohepatitis resolution (diagnostic category)** 9](#_Toc122690602)

[**Supplementary Figure 5. Digital quantitation of hepatocyte ballooning and co-localization analysis of ballooned hepatocyte and nearby fibrosis.** 10](#_Toc122690604)

[**Supplementary Figure 6. Mean change from baseline in fasting lipids, mmol/L (full analysis set). (A) Cholesterol; (B) Triglycerides; (C) Free glycerol; (D) Free fatty acid** 11](#_Toc122690605)

[**Supplementary Figure 7. Plasma concentrations of TXR and CVC during the study treatment period. (A) Pre-dose plasma concentrations for TXR; (B)** **Post-dose plasma concentrations for TXR; (C) Pre-dose plasma concentrations for CVC; (D) Post-dose plasma concentrations for CVC** 12](#_Toc122690606)

[**Supplementary Tables** 13](#_Toc122690607)

[**Supplementary Table 1. Resolution of steatohepatitis (FDA, EMA) without worsening of fibrosis (NASH CRN staging) – paired reading** 13](#_Toc122690608)

[**Supplementary Table 2. At least two-point improvement in fibrosis (NASH CRN) paired readings. (A) Regardless of steatohepatitis; (B) Without worsening of steatohepatitis** 13](#_Toc122690609)

[**Supplementary Table 3. Changes in NASH features on liver histology at Week 48 based on paired biopsy readings (full analysis set).** 14](#_Toc122690610)

[**Supplementary Table 4. Median change from baseline in insulin sensitivity measured by HOMA-IR (full analysis set)** 14](#_Toc122690611)

[**References** 14](#_Toc122690612)

# **Supplementary methods**

## **Exploratory study endpoints**

Exploratory study endpoints are reported elsewhere^1^ and include

- Changes in low-density lipoprotein cholesterol, high-density lipoprotein cholesterol, and fasting triglycerides from baseline to Week 24 and Week 48
- Change from baseline over time to Week 48 in the following parameters: Fasting insulin, glucose, and calculated HOMA-IR
- Change from baseline over time to Week 48 for anthropometric parameters, including body weight
- Change in magnetic resonance imaging-proton density fat fraction (MRI-PDFF) from baseline to Week 24 and Week 48
- Change in occurrence of potential itch based on a visual analog scale (VAS), from baseline to Week 24 and Week 48
- Free tropifexor (TXR) and cenicriviroc (CVC) serum concentrations over time (as calculated through C_max_, AUC, and other derived pharmacokinetic [PK] parameters)

## **Study assessments**

### **Liver biopsies**

Liver biopsies were performed using five to eight unstained liver biopsy sections prepared and submitted to the central pathologist who confirmed eligibility prior to randomization. If a suitable historical biopsy sample within 6 months prior to screening from which slides could be prepared was not available, the liver biopsy could be performed any time during the 10-week screening period.

### **Non-invasive tests**

A number of non-invasive tests were performed to assess liver damage and function. Liver steatosis (hepatic fat fraction) was quantified using MRI-PDFF. In addition, a transient elastography machine (Fibroscan^®^) was used to assess liver fat using controlled attenuation parameter scores (dB/m). Liver fibrosis was estimated using imaging assessments measuring liver stiffness (kPa): Magnetic Resonance Elastography (MRE) and Fibroscan®, and by blood-based assessments: the enhanced liver fibrosis (ELF) test, fibrosis biomarker test, and non-alcoholic fatty liver disease (NAFLD) fibrosis score.

### **PK analysis**

TXR and CVC were determined in plasma using a validated liquid chromatography coupled to tandem mass spectrometry method (LC-MS/MS). The lower limit of quantification in plasma samples was 20 pg/mL for TXR and 5 ng/mL for CVC. Pre-dose PK samples elapsed times were specified in the range ≥ ‒5 hours to <0.083 hours, post-dose samples with elapsed times ≥0.083 hours to 3.5 hours (average±SD post-dose elapsed time: 1.00±0.46 hours across study arms). These elapsed times ranges, which accounted for the majority of samples, were included in the summary statistics.

## **Post hoc digital pathology and artificial intelligence (AI) analyses of treatment-induced changes in steatosis, hepatocyte ballooning, and liver fibrosis**

Considering that NASH treatment with compounds that markedly reduce liver fat content can alter the area used for fibrosis quantitation, especially when comparing pre- and post-treatment fibrosis, a steatosis correction was applied when assessing fibrosis dynamics within the liver lobule. For this purpose, the steatosis area, as determined by SHG/TPEF microscopy, was subtracted from the total liver tissue area to ensure that the denominator is the same for quantitation of fibrosis in different zones of the liver lobule before and after treatment. The SHG/TPEF examination and analyses were performed blinded to clinical trial results, type of treatment, time point, and the NASH CRN scoring with the conventional microscopy.

# **Supplementary figures**

## **Supplementary Figure 1. Study design**

CVC, cenicriviroc; F, fibrosis stage; N, total number of patients; n, number of patients per group; NASH, non-alcoholic steatohepatitis; TXR, tropifexor


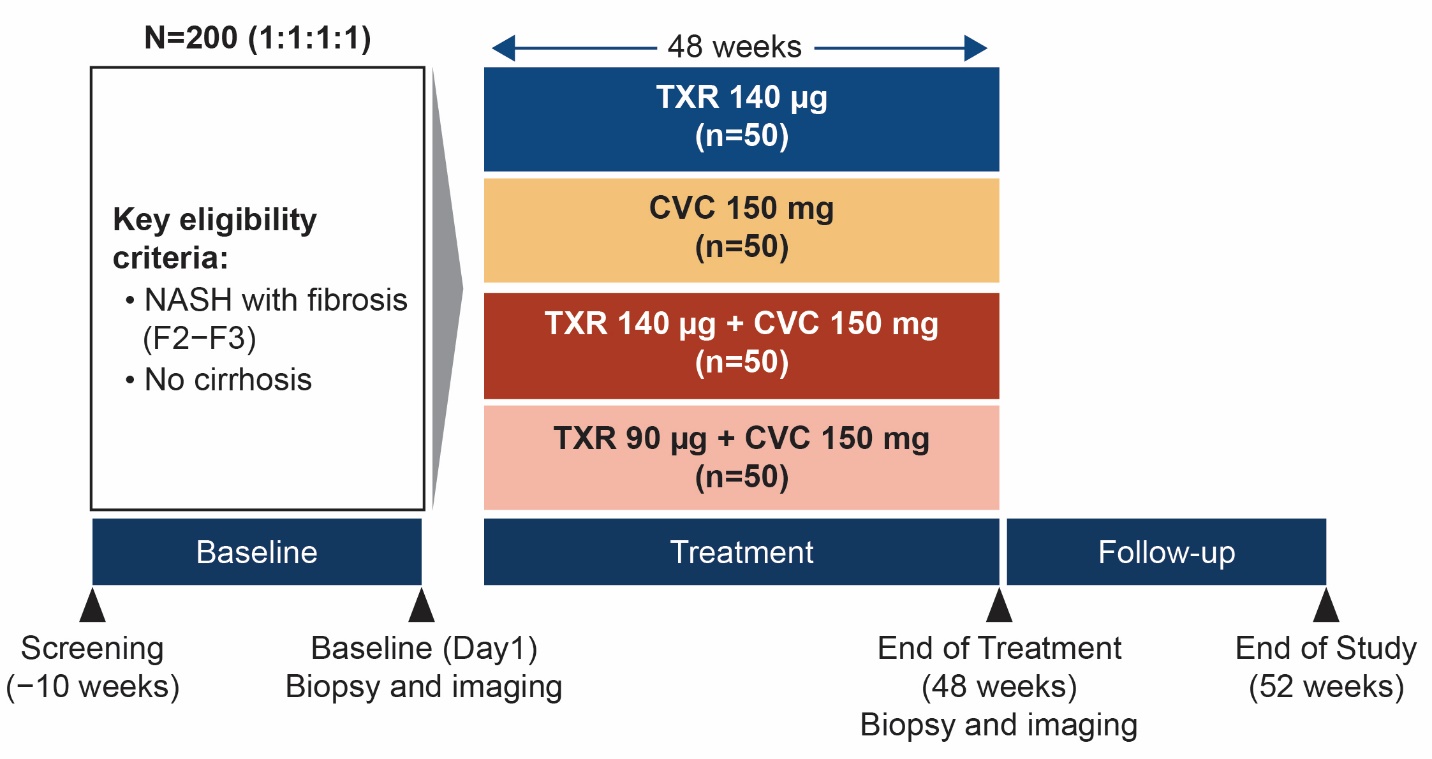


## **Supplementary Figure 2. Box plots for fibrosis markers. (A) Fibroscan; (B) MRE-liver stiffness; (C) ELF; (D) NAFLD fibrosis score; (E) Fibrosis biomarker test score**

Symbols: Box=1^st^‒3^rd^ (Q1‒Q3) quartiles; symbols inside each box=median; whisker length=largest value <1.5 (Q3‒Q1); symbols outside each box: outliers.

CVC, cenicriviroc; ELF, enhanced liver fibrosis; MRE, magnetic resonance elastography; NAFLD, non-alcoholic fatty liver disease; TXR_90_, tropifexor 90 µg; TXR_140_, tropifexor 140 µg.


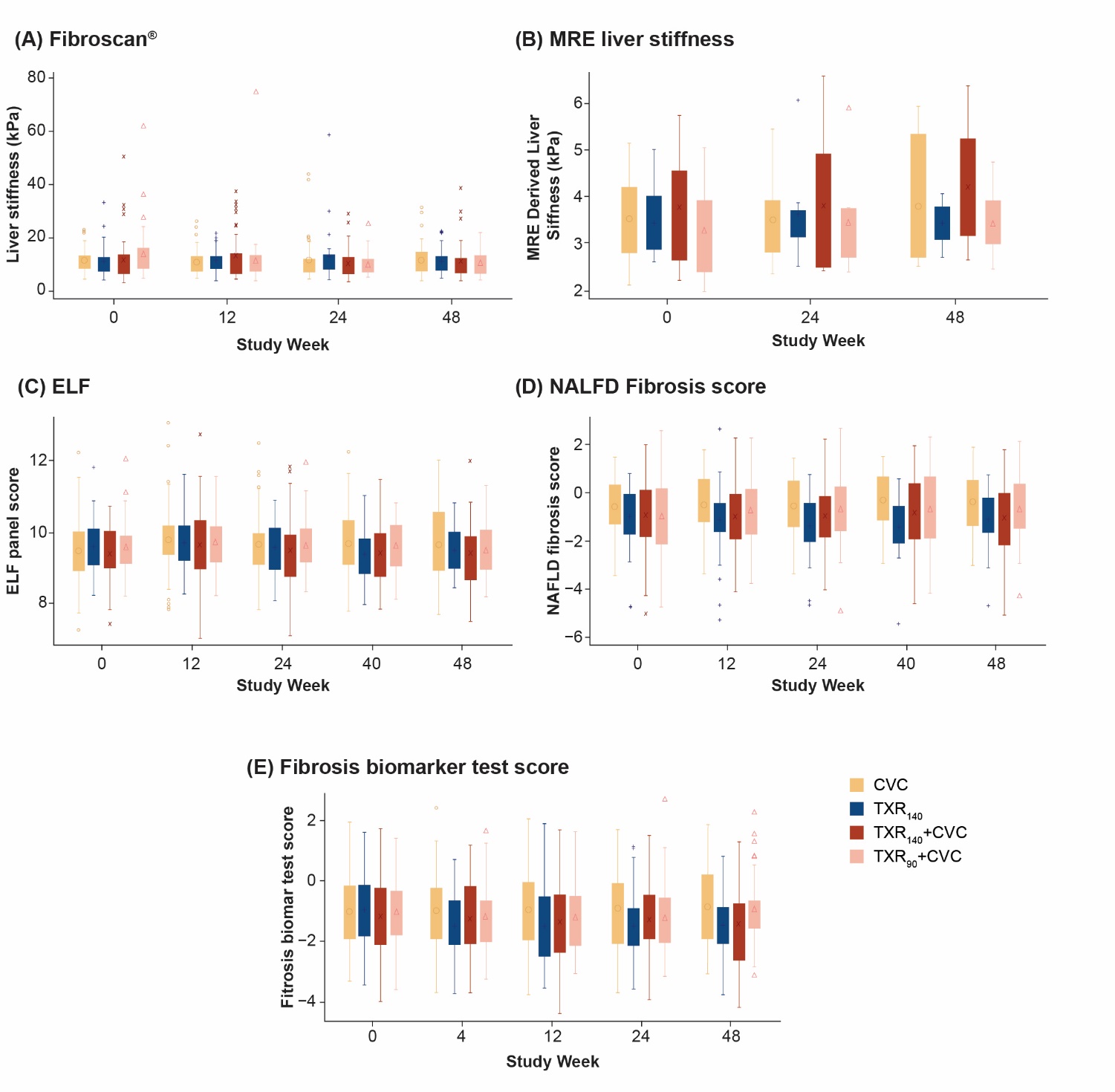


## **Supplementary Figure 3. Mean change from baseline in (A) FIB-4 and (B) High-sensitivity C-reactive protein**

CVC, cenicriviroc; FIB-4 fibrosis-4; HsCRP, high-sensitivity C-reactive protein; TXR_90_, tropifexor 90 µg; TXR_140_, tropifexor 140 µg


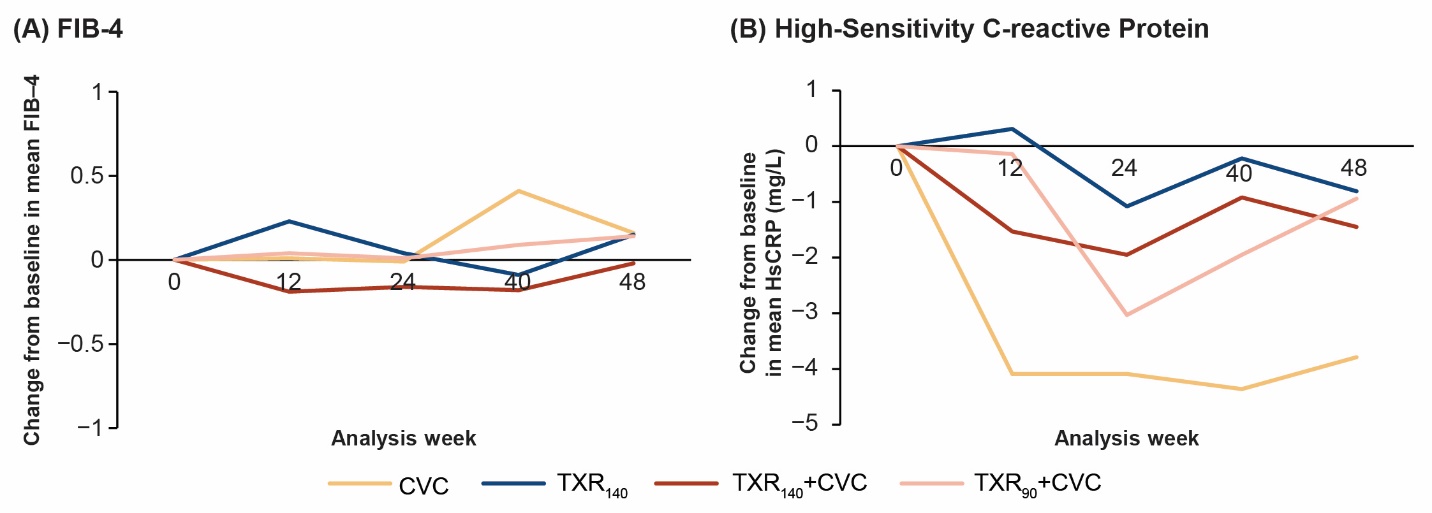


## **Supplementary Figure 4. Patients with histological responses at Week 48 in all groups based on NASH CRN staging: real-time reading. (A) Fibrosis improvement (≥1 point, NASH CRN staging); (B) Steatohepatitis resolution (diagnostic category)**

### CRN, clinical research network; CVC, cenicriviroc; NASH, non-alcoholic steatohepatitis; TXR_90_, tropifexor 90 µg; TXR_140_, tropifexor 140 µg


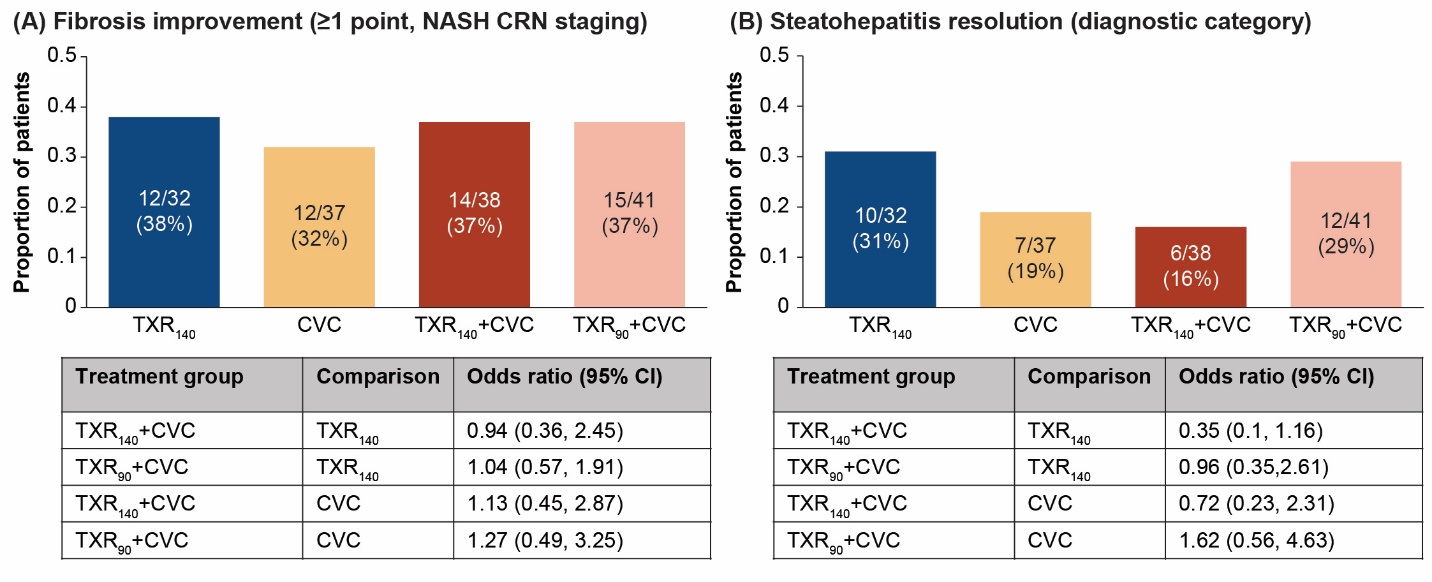


## **Supplementary Figure 5. Digital quantitation of hepatocyte ballooning and co-localization analysis of ballooned hepatocyte and nearby fibrosis.**

(A) Total number of ballooned hepatocytes in the liver biopsy (normalized per tissue area) across treatment groups; (B) Treatment-induced changes of ballooned hepatocytes and nearby fibrosis (subset of patients with “worsening” or “no change” in qBallooning grade from baseline to EOT); (C) Treatment-induced changes of ballooned hepatocytes and nearby fibrosis (subset of patients who had “improvement” in qBallooning grade from baseline to EOT)

CVC, cenicriviroc; EOT, end of treatment; n, number of patients per group; TXR_90_, tropifexor 90 µg; TXR_140_, tropifexor 140 µg


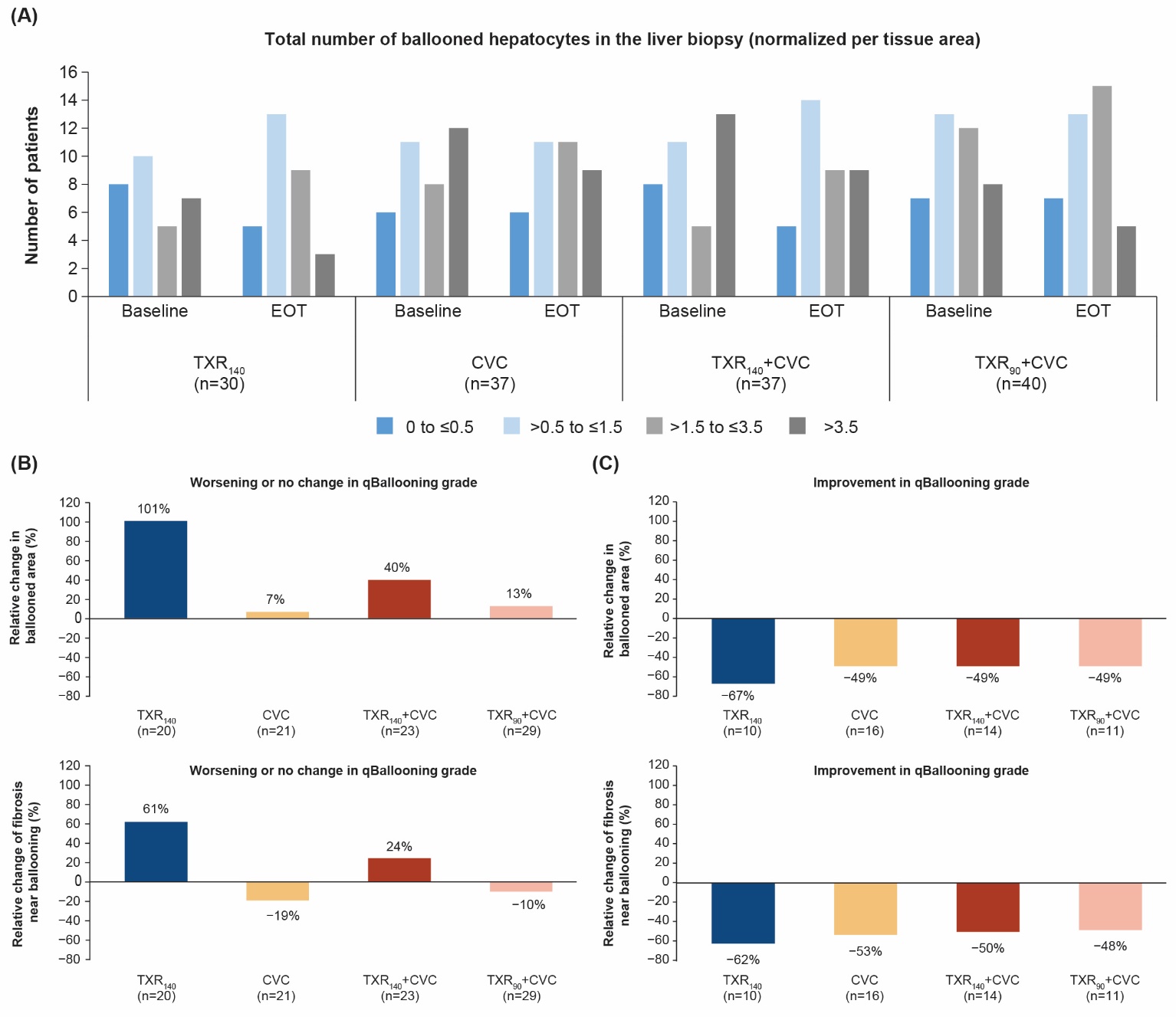


## **Supplementary Figure 6. Mean change from baseline in fasting lipids, mmol/L (full analysis set). (A) Cholesterol; (B) Triglycerides; (C) Free glycerol; (D) Free fatty acid**

CVC, cenicriviroc; TXR_90_, tropifexor 90 µg; TXR_140_, tropifexor 140 µg.


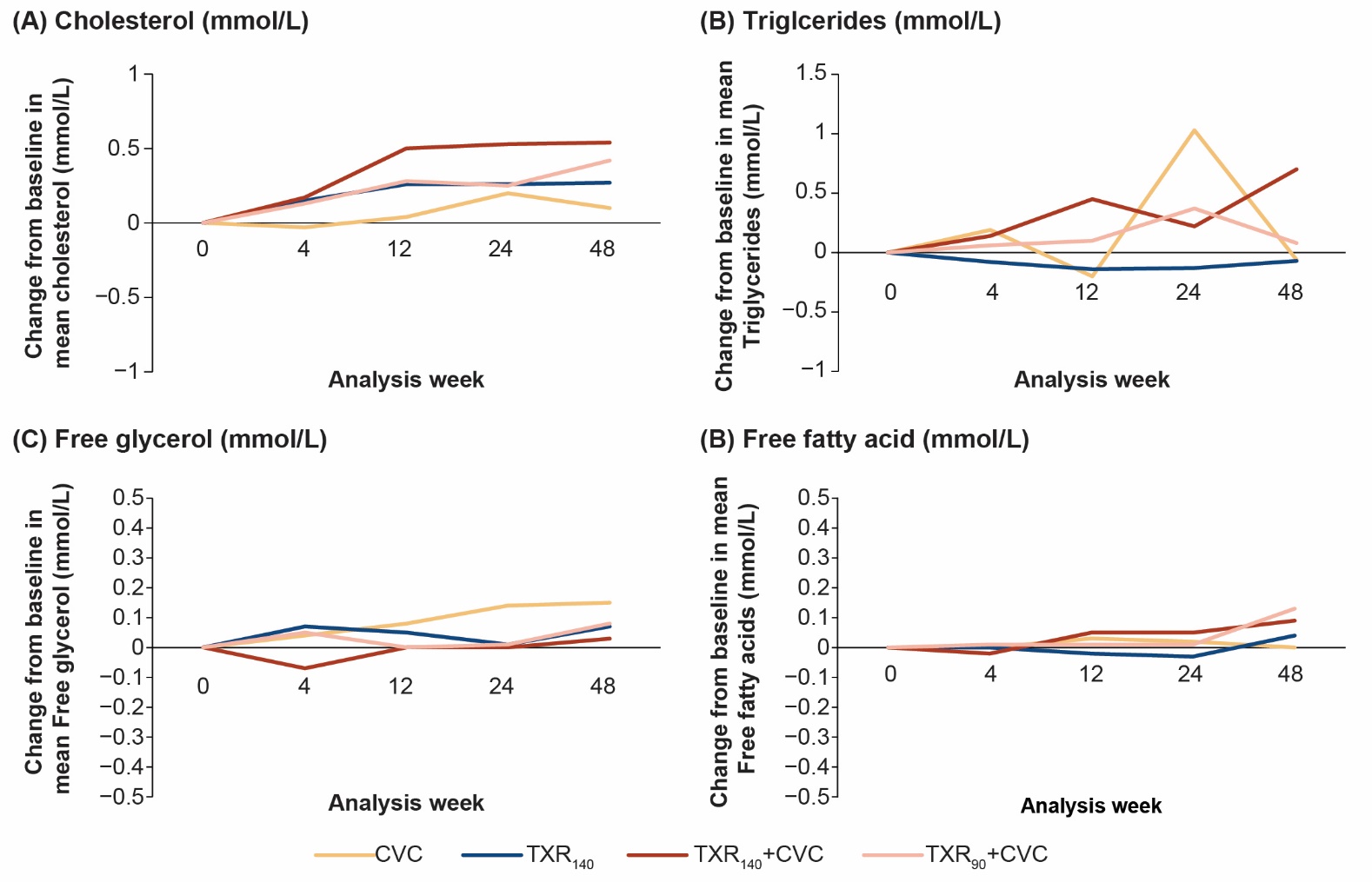


## **Supplementary Figure 7. Plasma concentrations of TXR and CVC during the study treatment period. (A) Pre-dose plasma concentrations for TXR; (B)** **Post-dose plasma concentrations for TXR; (C) Pre-dose plasma concentrations for CVC; (D) Post-dose plasma concentrations for CVC**

CVC, cenicriviroc; SEM, standard error of the mean; TXR_90_, tropifexor 90 µg; TXR_140_, tropifexor 140 µg


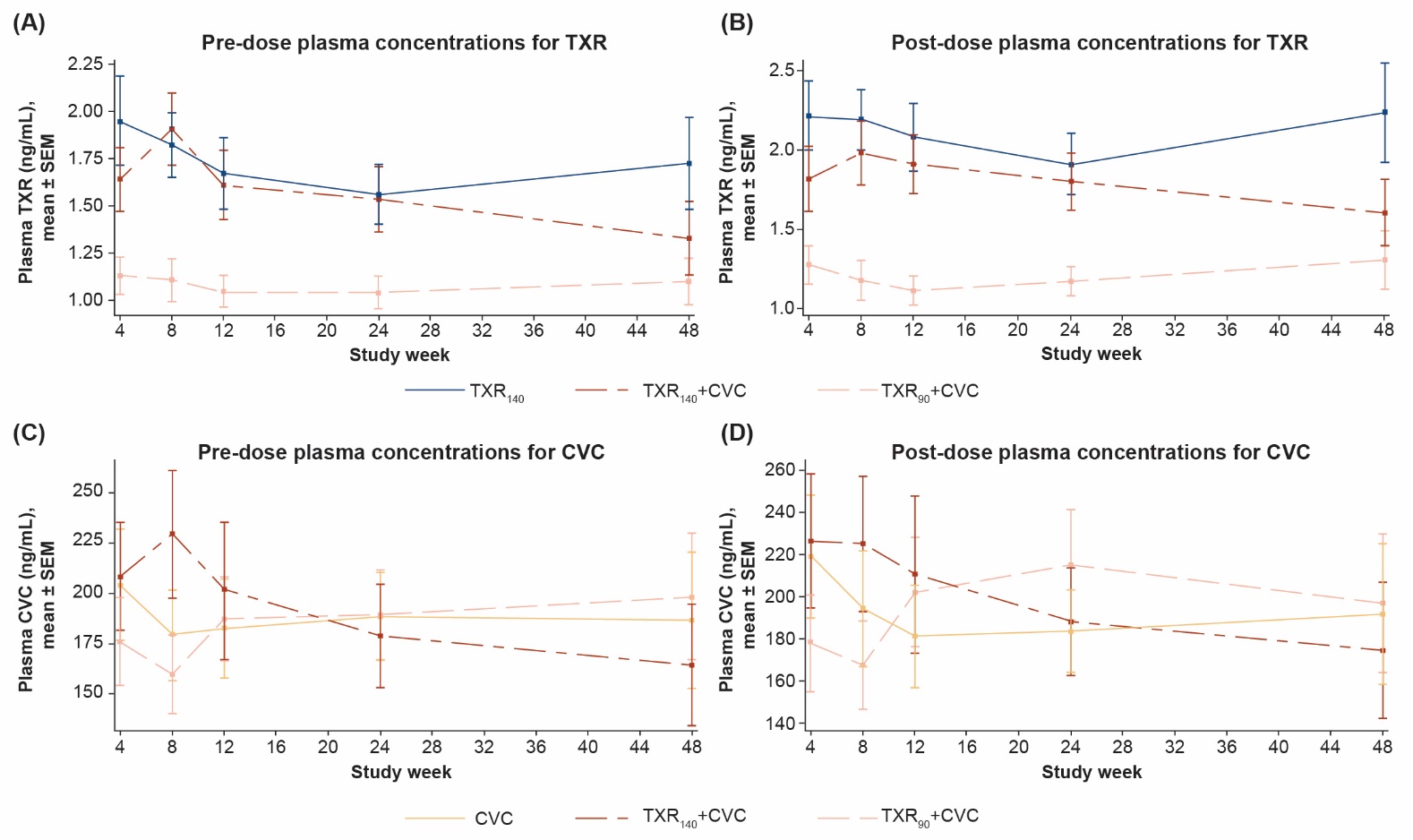


# **Supplementary Tables**

## **Supplementary Table 1. Resolution of steatohepatitis (FDA, EMA) without worsening of fibrosis (NASH CRN staging) – paired reading**

|  | **TXR_140_**  **N=50** | **CVC**  **N=48** | **TXR_140_+CVC**  **N=47** | **TXR_90_+CVC**  **N=48** |
| --- | --- | --- | --- | --- |
| **Resolution of steatohepatitis  (FDA, EMA) without worsening of fibrosis (NASH CRN staging) – paired reading, n/M (%)** | 5/31 (16) | 2/38 (5) | 1/37 (3) | 2/40 (5) |

FDA/EMA definition of resolution of steatohepatitis: Lobular inflammation=1 AND Hepatocyte ballooning=0 AND any value for steatosis.
CRN, clinical research network; CVC, cenicriviroc; EMA, European Medicines Agency; FDA, Food and Drug Administration; n, the number of patients who responded; N, the total number of patients in the treatment group; NASH, non-alcoholic steatohepatitis; M, the total number of patients with a value for a specific categorical variable; TXR_90_, tropifexor 90 µg; TXR_140_, tropifexor 140 µg

## **Supplementary Table 2. At least two-point improvement in fibrosis (NASH CRN) paired readings. (A) Regardless of steatohepatitis; (B) Without worsening of steatohepatitis**

1. **Regardless of steatohepatitis**

|  | **TXR_140_**  **N=50** | **CVC**  **N=48** | **TXR_140_+CVC**  **N=47** | **TXR_90_+CVC**  **N=48** |
| --- | --- | --- | --- | --- |
| **At least two-point improvement in fibrosis (NASH CRN staging) regardless of steatohepatitis status – n/M (%)** | 4/31 (13) | 7/38 (18) | 7/37 (19) | 4/40 (10) |
|  |  |  |  |  |

1. **Without worsening of steatohepatitis**

|  | **TXR_140_**  **N=50** | **CVC**  **N=48** | **TXR_140_+CVC**  **N=47** | **TXR_90_+CVC**  **N=48** |
| --- | --- | --- | --- | --- |
| **At least two-point improvement in fibrosis (NASH CRN staging)  without worsening of steatohepatitis – n/M (%)** | 4/31 (13) | 7/38 (18) | 6/37 (16) | 4/40 (10) |

CRN, clinical research network; CVC, cenicriviroc; n, the number of patients who responded; N, the total number of patients in the treatment group; NASH, non-alcoholic steatohepatitis; M, the total number of patients with a value for a specific categorical variable; TXR_90_, tropifexor 90 µg; TXR_140_, tropifexor 140 µg.

## **Supplementary Table 3. Changes in NASH features on liver histology at Week 48 based on paired biopsy readings (full analysis set).**

| **n/M (%)** | **TXR_140_**  **N=50** | **CVC**  **N=48** | **TXR_140_+CVC**  **N=47** | **TXR_90_+CVC**  **N=48** |
| --- | --- | --- | --- | --- |
| **At least one-point improvement in ballooning** | 8/31 (26) | 11/38 (29) | 7/37 (19) | 17/40 (43) |
| **At least one-point improvement in lobular inflammation** | 10/31 (32) | 8/38 (21) | 10/37 (27) | 11/40 (28) |
| **At least one point improvement in portal inflammation** | 8/31 (26) | 9/38 (24) | 12/37 (32) | 10/40 (25) |
| **Improvement in NAS score (at least 2 points with at least 1 point in >1 category)** | 6/31 (19) | 7/38 (18) | 7/37 (19) | 9/40 (23) |
| **Resolution of ballooning (score of 0)** | 6/31 (19) | 6/38 (16) | 4/37 (11) | 8/40 (20) |

CVC, cenicriviroc; n, the number of patients who responded; N, the total number of patients in the treatment group; NAS, NAFLD activity score; M, the total number of patients with a value for a specific categorical variable; TXR_90_, tropifexor 90 µg; TXR_140_, tropifexor 140 µg

## **Supplementary Table 4. Median change from baseline in insulin sensitivity measured by HOMA-IR (full analysis set)**

|  | **TXR­_140_**  **N=50** | **CVC**  **N=48** | **TXR­_140_+CVC**  **N=47** | **TXR_90_+CVC**  **N=48** |
| --- | --- | --- | --- | --- |
| **Week 24** | −0.78 | −0.16 | 0.68 | 0.80 |
| **Week 48** | −0.49 | 0.00 | 0.34 | 0.57 |

CVC, cenicriviroc; HOMA-IR, homeostatic model assessment for insulin resistance; TXR_90_, tropifexor 90 µg; TXR_140_, tropifexor 140 µg.

# **References**

1. Pedrosa M, Seyedkazemi S, Francque S, et al. A randomized, double-blind, multicenter, phase 2b study to evaluate the safety and efficacy of a combination of tropifexor and cenicriviroc in patients with nonalcoholic steatohepatitis and liver fibrosis: Study design of the TANDEM trial. *Contemp Clin Trials.* 2020;88:105889.
